# Isolation and functional characterization of *Lycopene β-cyclase *(*CYC-B*) promoter from *Solanum habrochaites*

**DOI:** 10.1186/1471-2229-10-61

**Published:** 2010-04-09

**Authors:** Monika Dalal, Viswanathan Chinnusamy, Kailash C Bansal

**Affiliations:** 1 National Research Centre on Plant Biotechnology, Indian Agricultural Research Institute, New Delhi - 110012, India; 2 Directorate of Sorghum Research, Hyderabad -500 030, India; 3 Water Technology Centre, Indian Agricultural Research Institute, New Delhi - 110012, India

## Abstract

**Background:**

Carotenoids are a group of C40 isoprenoid molecules that play diverse biological and ecological roles in plants. Tomato is an important vegetable in human diet and provides the vitamin A precursor *β*-carotene. Genes encoding enzymes involved in carotenoid biosynthetic pathway have been cloned. However, regulation of genes involved in carotenoid biosynthetic pathway and accumulation of specific carotenoid in chromoplasts are not well understood. One of the approaches to understand regulation of carotenoid metabolism is to characterize the promoters of genes encoding proteins involved in carotenoid metabolism. *Lycopene β-cyclase *is one of the crucial enzymes in carotenoid biosynthesis pathway in plants. Its activity is required for synthesis of both α-and β-carotenes that are further converted into other carotenoids such as lutein, zeaxanthin, etc. This study describes the isolation and characterization of chromoplast-specific *Lycopene β-cyclase *(*CYC-B*) promoter from a green fruited *S. habrochaites *genotype EC520061.

**Results:**

A 908 bp region upstream to the initiation codon of the *Lycopene β-cyclase *gene was cloned and identified as full-length promoter. To identify promoter region necessary for regulating developmental expression of the *ShCYC-B *gene, the full-length promoter and its three different 5' truncated fragments were cloned upstream to the initiation codon of *GUS *reporter cDNA in binary vectors. These four plant transformation vectors were separately transformed in to *Agrobacterium*. *Agrobacterium*-mediated transient and stable expression systems were used to study the *GUS *expression driven by the full-length promoter and its 5' deletion fragments in tomato. The full-length promoter showed a basal level activity in leaves, and its expression was upregulated > 5-fold in flowers and fruits in transgenic tomato plants. Deletion of -908 to -577 bp 5' to ATG decreases the *ShCYC-B *promoter strength, while deletion of -908 to -437 bp 5' to ATG led to significant increase in the activity of GUS in the transgenic plants. Promoter deletion analysis led to the identification of a short promoter region (-436 bp to ATG) that exhibited a higher promoter strength but similar developmental expression pattern as compared with the full-length *ShCYC-B *promoter.

**Conclusion:**

Functional characterization of the full-length *ShCYC-B *promoter and its deletion fragments in transient expression system *in fruto *as well as in stable transgenic tomato revealed that the promoter is developmentally regulated and its expression is upregulated in chromoplast-rich flowers and fruits. Our study identified a short promoter region with functional activity and developmental expression pattern similar to that of the full-length *ShCYC-B *promoter. This 436 bp promoter region can be used in promoter::reporter fusion molecular genetic screens to identify mutants impaired in *CYC-B *expression, and thus can be a valuable tool in understanding carotenoid metabolism in tomato. Moreover, this short promoter region of *ShCYC-B *may be useful in genetic engineering of carotenoid content and other agronomic traits in tomato fruits.

## Background

Carotenoids constitute a group of naturally occurring pigments that play diverse roles in plants. Structurally carotenoids are composed of eight isoprene units joined to form a C40 hydrocarbon skeleton containing conjugated double bonds and linear or cyclic end groups. In chloroplasts, they are part of light-harvesting complexes and also function as antioxidants. Carotenoids accumulate in chromoplasts as secondary metabolites, and impart attractive colors to flowers and fruits. They are important in human diet as they provide *β*-carotene, the vitamin A precursor. Owing to their antioxidant activity, they are also commercially used by cosmetic and pharmaceutical industries [1].

Carotenoid biosynthesis has been extensively studied in plants such as tomato, *Arabidopsis *and pepper [2]. Genes coding for enzymes catalyzing main steps of the carotenoid biosynthesis pathway have been cloned and their expression profiles have also been studied in different species [3-5]. Tomato fruit is a model system for studying the carotenogenesis in plants. Ripening in tomato fruit is associated with vivid changes in color. The change in fruit color from green to orange, pink and then red is accompanied by shift in carotenoid profile from β-carotene at breaker stage to lycopene at red ripe stage. These changes are brought about by transcriptional upregulation of *Phytoene Synthase *(*PSY1*) and *Phytoene Desaturase *(*PDS*) genes [6-9] and down regulation of *Lycopene β-cyclase *(*LCY-B*) and *Lycopene ε-cyclase *(*CRTL-e*) genes [9-12]. Significant increase in carotenoid content was achieved by genetic engineering of carotenoid biosynthesis pathway in canola, rice, potato and maize [13-17]. In contrast to these transgenic crops, only limited success has been achieved in increasing the carotenoid levels in tomato [18-21].

The carotenoid biosynthetic pathway is controlled by a complex regulatory mechanism that includes transcriptional, post transcriptional and feed-back inhibition by end-products [8,22-24]. Moreover, isoprene precursors required for the carotenoid pathway also serve as precursors for phytohormones such as abscisic acid (ABA), gibberellins and secondary metabolites [25,26]. Constitutive over-expression of chromoplast-specific PSY1 in tomato has been shown to result in dwarf phenotype, probably by interfering with the gibberellin biosynthesis pathway [27]. Contrary to the expectation, the PSY1 over-expressing transgenic tomato fruit also had reduced lycopene content as compared to untransformed plants [27]. Tomato *high-pigment 3 *(*hp3*) mutant showed 30% increase in carotenoid content in the mature fruit, but exhibited ABA deficiency [28]. Therefore, understanding the regulatory network and metabolic cross-talk between pathways is necessary for metabolic engineering of carotenoids in plants. The success of desired modification in transgenics depends upon the source of transgene; organ to which it is targeted, choice of promoter used, and the key nodes in pathway targeted for modification. The key steps in carotenoid biosynthetic pathway predominantly targeted for transgenic modifications are catalyzed by enzymes such as *PSY*, *PDS *and *LCY-B*. Although cDNAs of genes encoding carotenoid biosynthetic pathway enzymes have been well characterized in tomato, their promoters have received limited attention. Only *PDS *promoter has been characterized in tomato [8]. Isolation and characterization of promoters of carotenoid biosynthesis pathway genes will help understand the regulation of carotenoid biosynthesis pathway in tomato.

In this study, we describe the isolation and characterization of *CYC-B *(chromoplast-specific *lycopene β-cyclase*) promoter from a green fruited *Solanum habrochaites *genotype EC520061. The *CYC-B *full-length promoter and its 5' truncated promoter regions were analyzed by transient and stable expression systems using *GUS *as reporter gene in tomato. A short promoter region with higher expression level and developmental expression similar to that of full-length promoter was identified.

## Results and Discussion

Tomato genome contains two types of *lycopene β-cyclase *genes, *LCY-B *and *CYC-B*, encoding chloroplast- and chromoplast-specific *lycopene β-cyclase *enzymes, respectively. *LCY-B *is expressed in leaves, flowers and in fruits until breaker-stage of fruit ripening [10,12]. The *CYC-B *encodes a chromoplast-specific *lycopene β-cyclase *and is expressed exclusively in flowers and at breaker-stage of fruit [12]. *Lycopene β-cyclase *is one of the crucial enzymes for carotenoid biosynthesis. *Lycopene β-cyclase *along with *Lycopene ε-cyclase *(*LYC-E*) bring about the cyclization of lycopene. Activities of both of these enzymes together make α-carotene, while activity of *Lycopene β-cyclase *alone leads to formation of β-carotene [26]. In pepper also, *LYC-B *and *Capsanthin-Capsorubin Synthase *(*CCS*) genes have been proposed to bring about major changes in carotenoid profiles during ripening in pepper [5]. *CCS *is a protein with high homology to *CYC-B *of tomato, and is highly induced during fruit development [5,12]. In watermelon, co-dominant CAPS markers based on a SNP in *Lycopene β-cyclase *gene has been developed for allelic selection between canary yellow and red watermelon [29]. Thus, *lycopene β-cyclase *plays a major role in carotenogenesis of different colored flowers and fruits. Isolation and characterization of *CYC-B *promoter will help understand the regulation of *CYC-B *expression in chromoplast-rich organs, and the promoter may be useful in genetic engineering of carotenoid content in plants.

### Expression of *CYC-B* gene in *S. lycopersicum* and *S. habrochaites*

**Figure 1:**
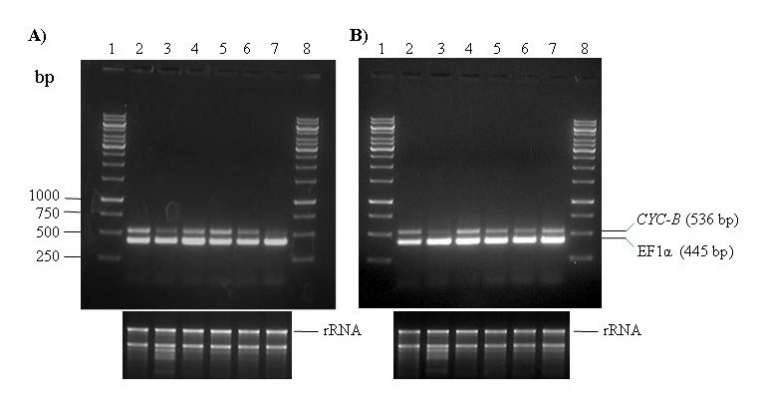
**Expression levels of *lycopene β-cyclase *(*CYC-B*) in different tissues of *S. lycopersicum and S. habrochaites***. RT-PCR was performed with total RNA isolated from leaves, flowers and fruits at different stages of development. (A) Wild-type tomato cv. Pusa Ruby (*S. lycopersicum*); Lanes 2-3, flower and leaf, respectively; Lanes 4-7; fruit at different stages of ripening, from mature green (lane 4), breaker (Lane 5), orange (lane 6) to red ripe stage (Lane 7). (B) *S. habrochaites *genotype EC520061. Lanes 2-3, flower and leaf, respectively; Lanes 4-7, fruit with progressive stages of ripening, from mature green (Lane 4) to ripe stage (Lane 7). In case of *S. habrochaites*, which is a green fruited genotype, progress of ripening was broadly defined on the basis of seed color and development (see Materials and Methods). Lanes 1 and 8, 1 kb Mol. weight marker.

The mRNA levels of *CYC-B *gene in leaf and flower, and at different stages of fruit development of *S. lycopersicum *cv. Pusa Ruby and *S. habrochaites *genotype EC520061 were determined by semi-quantitative RT-PCR analysis. Pusa Ruby fruits show typical color change from green to red during ripening, while *S. habrochaites *(EC520061) fruits remain green even at fully ripe stage. *CYC-B *expression was high in the chromoplast-rich tissues such as flowers and fruits, while a low basal level expression was detected in fully developed leaves in both the genotypes (Fig. 1). In Pusa Ruby fruits, *CYC-B *expression was highest in breaker stage, and thereafter reduced to a very low level at red-ripe stage. On the contrary, in *S. habrochaites *(EC520061) fruits, *CYC-B *expression levels were similar in different stages of fruit ripening, and remained high even at fully ripe stage. Expression pattern of *CYC-B *in *S. habrochaites *observed in this study was similar to that of *CYC-B *gene expression in *Beta *mutant reported by Ronen et al [12]. Although, Ronen et al [12] could not detect *CYC-B *expression in wild-type *S. lycopersicum *cv. M82, in this study we could detect expression of CYC-B in leaves, and in fruits at all the stages of ripening in both S. lycopersicum cv. Pusa Ruby and *S. habrochaites *genotype EC520061. *Expression pattern of CYC-B gene, a low level in leaves and high level in chromoplast-rich flowers and fruits, observed in our study is consistent with the expression patterns previously reported for PDS and PSY1 (*pTOM5) genes [6,8]. Similarly, *CrtR-b2 *(*β-carotene hyroxylase*), a chromoplast-specific gene is highly expressed in petals and anthers, and expresses albeit at low level in carpels and sepals, while *CrtR-b1*, a chloroplast-specific gene shows high level of expression in leaves and sepals but show a low level of expression in flower tissues [24]. Moreover, it was shown that transgenic plants expressing antisense *B *(*Beta*) did not show any biochemical or developmental alterations in leaves and stems [12]. Thereby implicating that the basal expression level of *CYC-B *found in leaves may not have critical role in vegetative tissues.

### Isolation of *CYC-B* promoter

**Figure 2:**
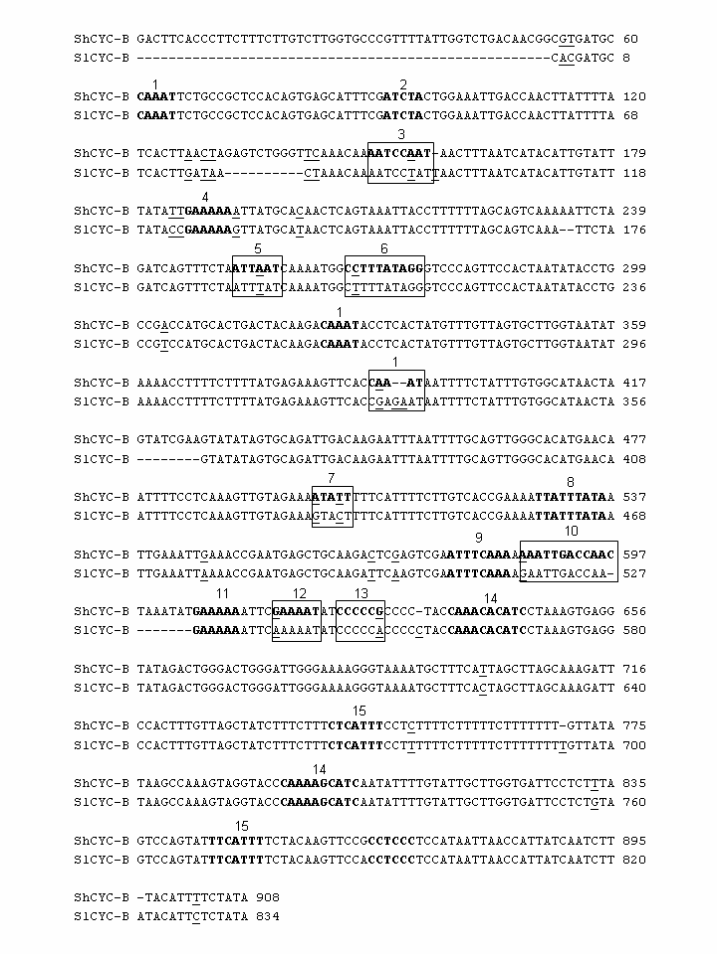
**Sequence comparison and putative *cis*--elements identified in the *CYC-B *promoter from *S. habrochaites *and *S. lycopersicum***. The *cis*-elements are numbered 1-15, *cis*-elements shared by the promoters are shown in bold; elements that are exclusive in one of the promoters are shown in boxes, difference in nucleotide is underlined. 1) CAAT Box; 2) RAP2.2; 3) rbcS consensus sequence; 4) GT1 CONSENSUS; 5) conserved DNA module for light responsiveness; 6) CARGAT CONSENSUS; 7) ROOT MOTIF TAPOX1;8) TATA box; 9) ERE LEE4; 10) L-box of *S. lycopersicum*, part of a light responsive element; 11) GT1 GM SCAM4; 12) GT1CONSENSUS; 13) GC-motif; 14) CIACADIANLELHC; 15) INRNTPSADB; The 5' upstream sequence of *CYC-B *gene is submitted at NCBI [*ShCYC-B *and *SlCYC-B *promoter GenBank accession numbers are DQ858292 and EU825694, respectively]. The *cis*-elements are described in detail in Table 1.

The promoter region of *CYC-B *gene from *S. habrochaites *genotype EC520061 and *S. lycopersicum *genotype EC521086 was isolated by directional genome walking PCR using a set of walker primers and gene-specific primers. Comparison of the sequence of the DNA fragment cloned from *S*. *habrochaites *with the coding sequence of *CYC-B *coding sequence [GenBank: AF254793] revealed the presence of 908 bp fragment upstream to the start codon of *CYC-B *gene. The 908 bp fragment upstream to the ATG codon was designated as *ShCYC-B *full-length promoter (Fig. 2). The nucleotide sequence of *ShCYC-B *promoter cloned in this study was deposited at NCBI [GenBank: DQ858292]. The DNA fragment cloned from *S. lycopersicum *contained 834 bp promoter region [GenBank: EU825694]. The *ShCYC-B *promoter sequence was analyzed for transcription start site (TSS) and potential *cis*-acting transcription factor binding sites. Neural Network Promoter Prediction [30] identified a TSS at 303 bp 5' to ATG. PLACE [31] and PlantCARE [32] analysis revealed a potential TATA box at 381 bp 5' to ATG and 72 bp upstream to the potential TSS. Several *CAAT *boxes, a RAP2.2 transcription factor binding site, an ethylene responsive element (*ERE*), circadian elements and light responsive elements were found in the *ShCYC-B *promoter (Fig. 2). A list of some of the relevant *cis*-elements detected and their relative positions from the translational start site ATG is given in Table 1. A comparison of the *CYC-B *promoters from *S. lycopersicum *and *S. habrochaites *revealed that *ERE *(*ATTTCAAA*) *cis*-element was conserved between these two promoters (Fig. 2). This *ERE cis*-element was also reported in *CYC-B *promoter of *Beta *gene [12]. This indicates ethylene responsive regulation of *CYC-B *promoter during fruit ripening. The RAP2.2 transcription factor binding site *ATCTA *[33] was also found in both the *CYC-B *promoters cloned in this study. The *cis-*element *ATCTA *has been found to be conserved in promoters of genes involved in carotenoid and tocopherol biosynthesis, and certain photosynthesis-related genes in *Arabidopsis *[33,34]. However, there were many elements that were exclusively present in *ShCYC-B *promoter but not in *SlCYC-B *promoter. These include *rbcS *general consensus sequence, CArG consensus sequence found in the promoter of flowering-time gene (*At SOC1*), L-Box (a part of light responsive element) and GT-1 element, which are known to play important role in gene expression (Table 1).

**Table 1:** List of *cis*-elements identified in 908 bp *ShCYC-B *promoter sequence

Name of *Cis*-element	Sequence	Position from ATG	Description
RAP2.2	ATCTA	-817	RAP2.2 cis-element is conserved in promoters of genes involved in carotenoid and tocopherol biosynthesis, and certain photosynthesis- related genes in Arabidopsis. It confers strong basal activity to promoter
rbcS consensus sequence	AATCCAA or AATCCAAC	-759	Influences the level of gene expression and involved in light regulated gene expression
GT1 consensus	GAAAAA	-224,-230, -294, -304, -386, -413, -557, -723, -809	Consensus binding site in many light-regulated genes, GT-1 can stabilize the TFIIA-TBP- TATA box complex
GT1 GM SCAM4	GAAAAA	-304, -723	GT-1 motif found in the promoter of soybean, Interacts with a GT-1-like transcription factor
CArGAT consensus	CCWWWWWWGG	-643	*Cis*-element found in the promoter of *AtSOC1*, a MADS-box flowering-time gene. Flowering Locus C (FLC) protein binds to GArG box in *SOC1 *promoter and represses the expression of *SOC1*.
ROOT MOTIF TAPOX1	ATATT	-104,-409,-728	Motif found in promoters of rolD and root-specific genes
ERE	AWTTCAAA	-332	Ethylene responsive element found in tomato E4 promoter and other senescence associated gene promoters. It is required for ethylene-mediated expression.
L-box	AAATTAACCAAC	-323	Part of a light-responsive element
CIACADIANLELHC	CAANNNNATC	-115, -273	Region necessary for circadian expression of tomato Lhc gene
INRNTPSADB	YTCANTYY	-64, -168, -403	Inr (initiator) elements found in tobacco psaDb promoter without TATA boxes, element responsible for light responsive transcription

### Transient and stable expression of *ShCYC-B* promoter in tomato

**Figure 3:**
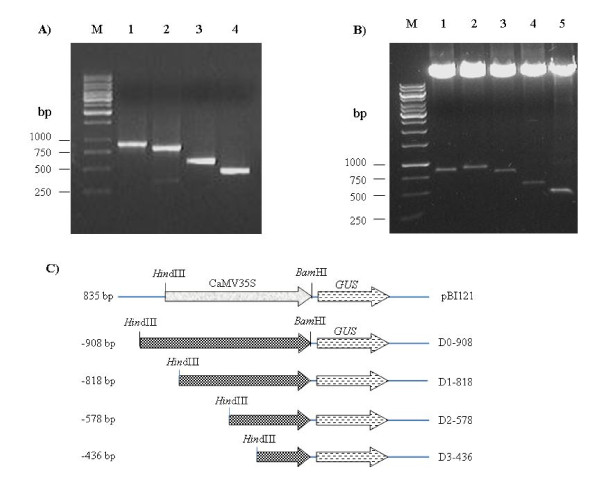
**Cloning of *ShCYC-B *full-length promoter and its deletion fragments in binary vector**. (A) PCR amplification of full-length and 5' deletion fragments of *ShCYC-B *promoter. M, 1 kb Mol. wt. marker; Lanes 1-4, amplicons of 908 bp, 818 bp, 578 bp and 436 bp, respectively. (B) Restriction confirmation of cloning of full-length and deletion fragments of *ShCYC-B *promoter in binary vectors. M, 1 kb Mol. wt. marker; Lanes 1-5, plasmids pBI121, pD0-908, pD1-818, pD2-578 and pD3-436, respectively, restricted with *Hind*III and *Bam*HI. (C) Schematic illustrations of *ShCYC-B *promoter and its deletion fragments. The numbers on the left indicate the 5' end points of the promoter fragments relative to the translational start site. Binary vector pBI121 having *GUS *gene driven by *CaMV35S *promoter was used as a positive control.

**Figure 4:**
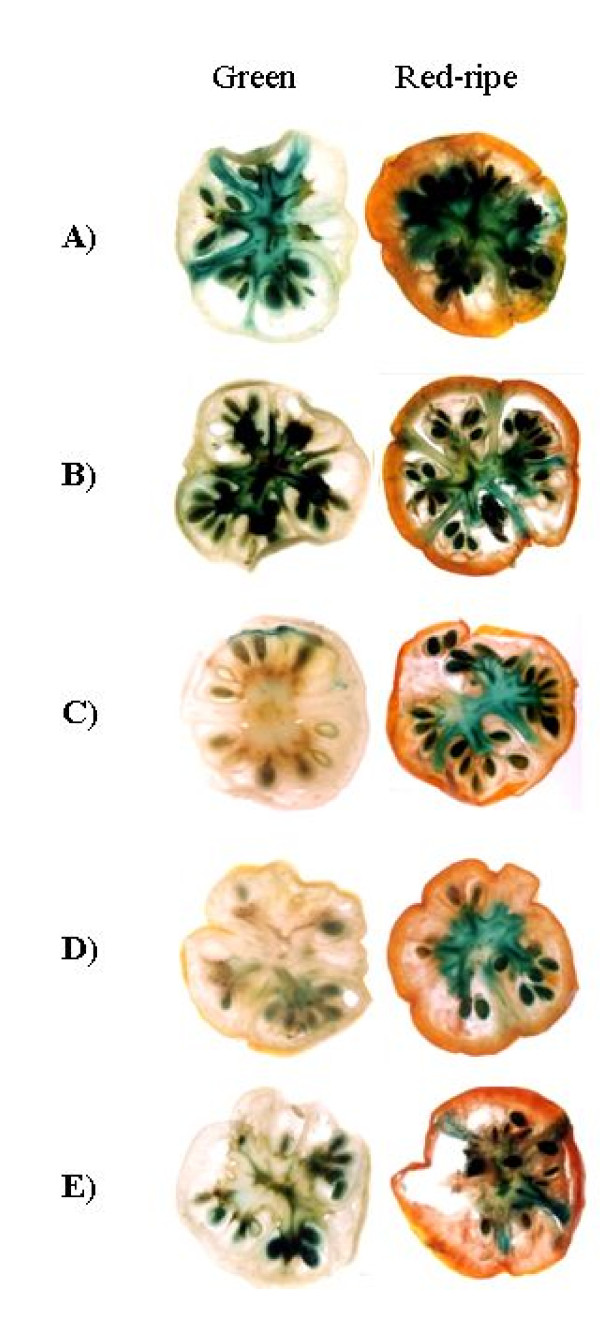
**Transient *in fruto *expression analysis of promoter::*GUS *activity in tomato**. (A) P_*CaMV*35*s*::_*GUS *(constitutive expression), (B) *ShCYC-B *D0-908::*GUS*, (C) pD1-818::*GUS*, (D) pD2-578::*GUS*, and (E) pD3-436::*GUS*. Tomato fruits were agro-injected with the promoter::*GUS *constructs, and GUS histochemical staining was performed on third day following agroinjection.

To characterize the putative *ShCYC-B *promoter, promoter::β-glucuronidase (*GUS*) reporter gene fusion constructs were prepared for full-length and its 5' deletion fragments, and transformed into *Agrobacterium *(Fig. 3). Binary vector pBI121 having constitutive *CaMV35S *promoter::*GUS *reporter gene was used as control. Functional analysis was carried out by transient *in fruto *expression in tomato fruits, and stable expression in transgenic tomato. In transient expression analysis, GUS activity was evident in columella and placental tissues in both green stage and ripe stage of tomato fruit (Fig. 4). This qualitative GUS assay revealed that the full-length *ShCYC-B *promoter as well as its deletion fragments were able to drive the expression of reporter gene in different developmental stages of tomato fruit.

To identify putative *cis*-elements required for developmental and tissue-specific expression of *ShCYC-B *promoter, tomato transgenic plants were generated. Six to nine independent transgenic lines for each construct were screened in T_0 _generation on the basis of PCR and histochemical GUS staining. There was no difference in the localization of GUS activity in fruits among full-length and truncated promoter constructs at various stages of fruit development. GUS staining was visible in vascular bundles, columella, placental tissue and seeds. In case of D0-908 (full-length *CYC-B *promoter) and D3-436 (the shortest promoter fragment) lines, locular tissue was also highly stained. However, there was no GUS activity in epidermis. The intensity of GUS staining was relatively similar among independent events for each construct, though one or two events of D1-818 and D2-578 transgenics showed variation in the intensity of GUS stain. Among the 9 independent events examined for D2- 567 transgenic plants, 7 plants showed consistently lower GUS intensity as compared to that of full-length and other deletion constructs (data not shown). About 4-5 individual events for each construct were carried forward to T_1 _for further analyses.

**Figure 5:**
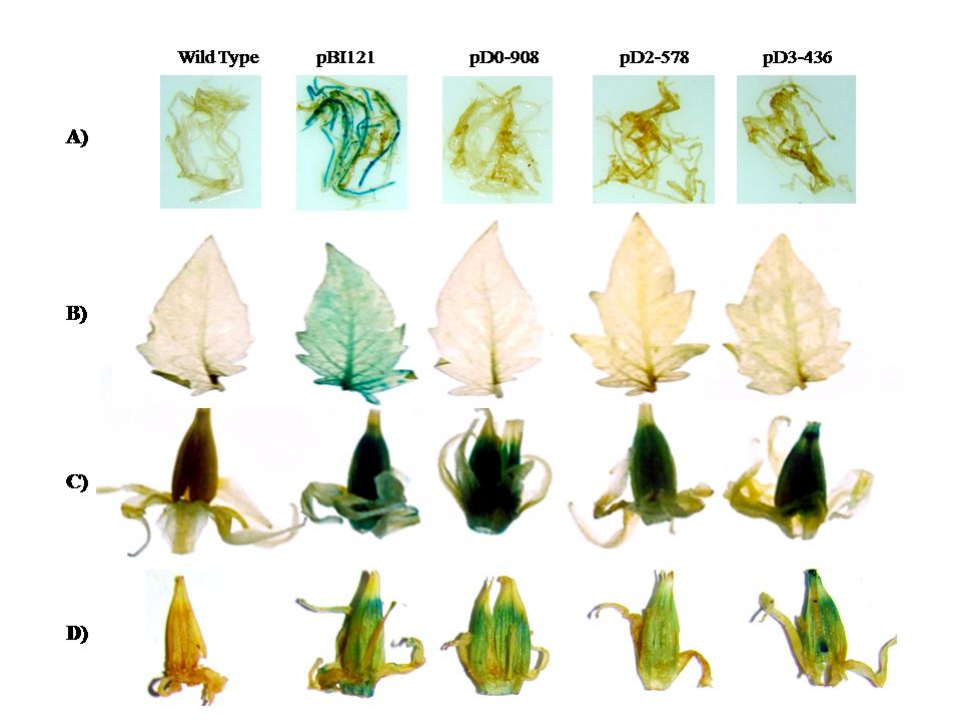
**Histochemical analysis of *ShCYC-B *full-length and its deletion fragments in transgenic tomato**. (A) Roots, (B) Leaves, and (C) Flowers. (D) Longitudinal section of flowers showing GUS staining mainly in stamens. pBI121 having *GUS *gene driven by *CaMV35S *promoter was used as a positive control. Wild type leaf served as negative control; D0-908, D2-578, and D3-436 represent transgenic plants carrying *ShCYC-B *full-length promoter D0-908::*GUS *and its deletion fragments D2-578::*GUS *and D3-436::*GUS *respectively.

**Figure 6:**
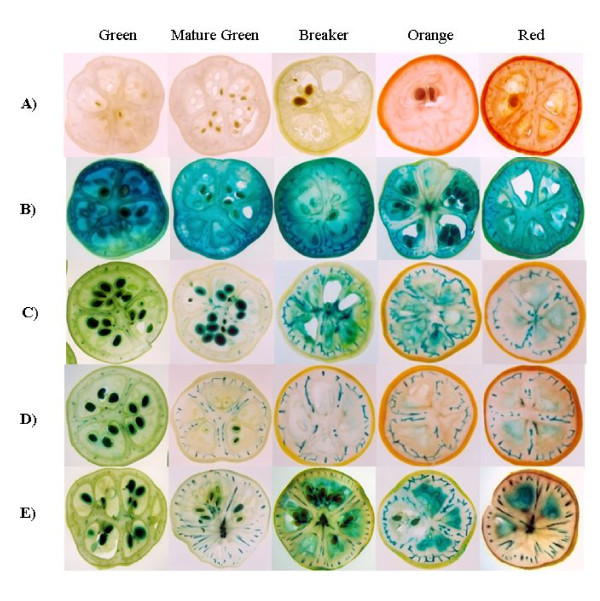
**Histochemical analysis of *ShCYC-B *full-length and its deletion fragments in tomato fruits**. *GUS *expression in (A) wild-type fruits, (B) transgenic fruits carrying P_*CaMV*35*S*::_*GUS *(constitutive promoter), and (C-D) transgenic fruits carrying *ShCYC-B *full-length promoter D0-908::*GUS *and its deletion fragments D2-578::*GUS *and D3-436::*GUS*, respectively in early green, mature green, breaker, orange and red ripe stages of fruit ripening. The images of different stages of fruits were derived from one representative line harboring single copy of transgene for each *ShCYC-B *promoter construct.

T_1 _seeds were selected on kanamycin and screened by PCR. For each construct, 2-3 events were subjected to Southern analysis to determine transgene copy number (Additional file 1, Fig. S1). Subsequently, plants having single copy insertions were analyzed for promoter activity by northern analysis, and histochemical as well as quantitative GUS assays. Since there was no visible difference in GUS staining of D0-908 and D1-818 transgenic fruits, and no single copy T_1 _plants were available in D1-818, plants harboring this construct were not included in further analysis. To examine the tissue-specific expression, leaf, root, flower and fruits at different developmental stages from single copy transgenic plants for each construct were subjected to histochemical GUS staining. GUS activity was apparently not detectable by visual observations in transgenic roots (Fig. 5A) and leaves (Fig. 5B) transformed with the *ShCYC-B *full-length or truncated promoter constructs. The transgenic flowers harboring either full-length *ShCYC-B *promoter or its 5' deletion fragments showed GUS staining mainly in stamens, while there was little or no GUS staining in petals (Fig. 5C-D). Similar kind of GUS staining was observed in the flowers of transgenic tomato expressing *PDS *promoter-driven *GUS *reporter gene [8]. The *CaMV35S::GUS *transgenic plants showed GUS staining both in leaves and flowers (Fig. 5). In T_1 _fruits, localization of GUS activity was similar to that observed in T_0 _fruits for all *ShCYC-B *promoter constructs. In fruits, D2-578 consistently showed lower GUS intensity, while the GUS staining was highest in D3-436 (the shortest promoter fragment). The activity of full-length and truncated promoter driven GUS was low at green fruit stage, and showed an upregulation at breaker and orange stages of fruit development (Fig. 6). Since *ERE *(ATTTCAAA) *cis*-element was found at -332 bp 5' to ATG of *ShCYC-B *promoter, we examined whether ethylene could induce the expression of *CYC-B *promoter in vegetative tissues. Foliar spray of Ethephon (1-5%) did not induce *CYC-B *promoter in the seedlings of full-length and deletion transgenic lines (data not shown). This suggests that developmental (flower and fruit) cues are required to induce CYC-B promoter.

### Quantitative GUS assay

**Figure 7:**
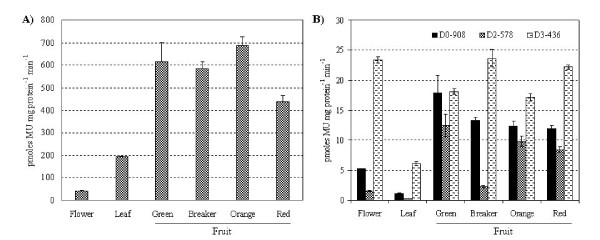
**Fluorometric quantification of GUS activity in transgenic tomato plants**. (A) Tissues from transgenic plants harboring P_*CaMV*35*S*::_*GUS *(constitutive promoter), (B) *ShCYC-B *full-length promoter D0-908::*GUS *and its deletion fragments D2-578::*GUS *and D3-436::*GUS*, respectively. Samples from 2-3 lines from single copy transgenic events (two events per construct) were pooled and used for quantitative MUG assay. Plant tissues were homogenized in protein extraction buffer. The supernatant was used for protein quantification and fluorometric assay. Reading at zero time point served as a control. The amount of 4-MU was determined from a standard curve. GUS activity was expressed as pmol 4-MU mg protein^-1 ^min^-1^. Data are presented as the mean (± standard error) of GUS activity from three independent determinations.

The visual observations made by histochemical GUS staining in leaf, flower and different developmental stages of fruit, were quantified by fluorometric MUG (4-methylumbelliferone glucuronide) assay. Tomato transgenic plants harboring pBI121 (constitutive *CaMV35S *promoter-driven GUS) were also included in the analysis. *CaMV35S *promoter driven GUS activity was lowest in flowers, and increased about 4- and > 8-fold in leaves and fruits, respectively (Fig. 7A). The expression pattern of full-length *ShCYC-B *promoter as determined by GUS activity (Fig. 7B) was similar to that of the expression pattern observed by RT-PCR analysis in *S. habrochaites *(Fig. 1B). In full-length *ShCYC-B *promoter transgenic plants, GUS activity was about 5- and > 12-fold higher in flower and fruits, respectively, as compared with leaves. Similarly, *ShCYC-B *D2-578 and D3-436 promoter transgenic plants also showed lower GUS activity in leaves as compared to flower and fruits (Fig. 7B). It appears that *ShCYC-B *promoter activity in leaves is too low to be detected by histochemical GUS staining. The D2-527 promoter transgenic plants consistently showed lowest GUS activity as compared to full-length and D3-436 truncated-promoter transgenic plants in all the tissues. Although the promoter strength of D3-436 was similar to that of full-length promoter in green fruits, D3-436 promoter showed higher activity than full-length promoter in leaves, flowers, and breaker-, orange- and red-stages of fruits (Fig. 7B). Most noticeably, the shortest promoter fragment D3-436 showed 4.5 and 5.11-fold higher GUS activity in flowers and leaves, respectively, as compared to that of D0-908 full-length promoter (Fig. 7B).

### Northern-blot analysis

**Figure 8:**
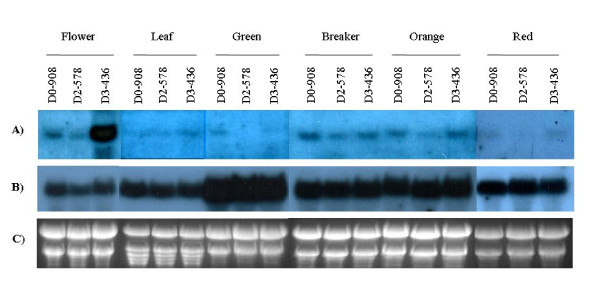
**Northern-blot analysis of *GUS *reporter expression in transgenic tomato plants transformed with full-length *ShCYC-B *promoter and its deletion fragment constructs**. (A) Expression of *GUS *gene driven by full-length *ShCYC-B *promoter and its deletion fragments D2-578::*GUS *and D3-436::*GUS*, respectively, (B) expression of *EF1 α *and (C) total RNA loaded per lane in the gel for northern blotting. Total RNA was isolated from the leaf, flower, and fruit tissue at green, breaker, orange and red ripe stage, from single copy transgenic events of each construct. RNA (20-30 μg) was electrophoresed on a 1.2% (w/v) formaldehyde agarose gel, transferred onto nylon membranes, and probed with ^32^P-dCTP-labeled *GUS *gene fragment. The same blot was reprobed with *EF1α *and was used as RNA loading control. The results shown are from tissues of one representative line harboring single copy of transgene for each *ShCYC-B *promoter construct.

Northern blot was performed to analyze the relative levels of *CYC-B *full-length or truncated promoter driven *GUS *expression in different tissues namely leaf, flower and fruits from transgenic tomato plants. Blots used for detecting *GUS *mRNA levels were reprobed with *EF1α *that served as a loading control for amount of total RNA. The concentration of total RNA loaded per lane for a particular stage of development or tissue was essentially same for transgenic lines of each construct, but was varying in the range of 20-30 μg across different stages or tissues used. *ShCYC-B *full-length promoter and its deletion fragments showed low expression in chloroplast-rich green leaves and green fruits, while their expression was high in chromoplast-rich flowers, and fruits at breaker and orange stage of ripening (Fig. 8). Consistent with GUS assay, in northern analysis also, D3-436 promoter fragment showed highest promoter activity in flowers (Fig. 8). However, in contrast to the higher GUS-activity observed in fruits of transgenic plants expressing D3-436 promoter driven *GUS *reporter, the transcript levels of D3-436 promoter driven *GUS *were not so apparently higher than that of D0-908 promoter driven *GUS *reporter. The deletion fragment D2-578 showed lowest promoter strength as compared to the full-length promoter and D3-436. The reduction in *GUS *expression in D2-578 transgenic plant does not appear to be due to transgene position and/or silencing effect, as seven out of nine independent events showed reduced GUS staining, and the transgenic plants with single insertion for D2-578 fragment were selected for analysis.

The present study clearly showed that the *ShCYC-B *promoter is developmentally regulated and its expression is upregulated in chromoplast-rich flowers and fruits at different stages of ripening. The promoter strength was drastically decreased by a deletion of -908 to -568 bp 5' to ATG as compared to full-length promoter, while the shortest D3-436 promoter fragment showed highest activity. This suggests that nucleotide sequence -567 to -437 bp upstream to initiation codon may contain *cis*-elements involved in down regulation of *CYC-B *expression, while nucleotide sequences -908 to -568 bp upstream to initiation codon might be involved in negating the repressive nature of regulatory sequences in -567 to -434 bp 5' to ATG. The RAP2.2 binding *cis*-element ATCTA has been shown to confer strong basal activity in *PSY *promoter from *Arabidopsis *[33]. This *cis-*element was found in *ShCYC-B *full-length promoter at -817 to -813 bp upstream to ATG, and might contribute to the basal activity of full-length promoter, as deletion of this *cis*-element in D2-578 resulted in considerable decrease in promoter activity. The presence of three GT-1 *cis*-element (GAAAAA), one at -723 to -718 and two at -304 to -299 and -294 to -289 bp upstream of ATG might be playing a role in the gene expression. GT-1 element was initially identified as *cis*-element regulating cell-type specific expression specifically in light regulated genes. GT-1 protein binds to TFIIA and TATA-binding proteins. The GT-1 *cis*-element is conserved in many plant promoters, and may have positive or negative regulatory effect on transcription depending upon cell type [35,36].

## Conclusion

In this study, Lycopene β-cyclase (*CYC-B*) promoter from *Solanum habrochaites*, a green fruited tomato genotype, was isolated and functionally characterized in transient *in fruto *and stable transformation systems. Conservation of *cis*-elements such as RAP2.2 binding *cis*-element and *ERE cis*-element between *CYC-B *and *PSY *promoter suggests a common regulatory mechanism for carotenoid accumulation in fruits. A short promoter region with promoter activity and developmental expression pattern comparable to that of full-length *ShCYC-B *promoter was identified. As signal transduction events and transcription factors that developmentally regulate the *CYC-B *expression are not known, the short promoter region identified in this study can be used in promoter::reporter fusion molecular genetic screens to identify mutants impaired in *CYC-B *expression, and thus can be a valuable tool in understanding carotenoid metabolism in tomato.

## Methods

### Isolation and cloning of *ShCYC-B* promoter

Isolation of 5' flanking region of *CYC-B *gene from *S. lycopersicum *genotype EC521086 and *S. habrochaites *genotype EC520061 was carried out following PCR-based directional genome walking method [37]. Genomic DNA was extracted from leaves following cetyl trimethyl ammonium bromide (CTAB) method [38]. In the primary PCR, genomic DNA was used as template. Amplification was carried out with biotinylated gene specific reverse primer (R1) along with one of the four universal walker primers namely Walker 1, Walker 2, Walker 3 and Walker 4 (Table 2) in four different reactions. The PCR conditions were as follows: initial denaturation at 94°C for 4 min followed by 33 cycles of 94°C (1 min), 47°C (1 min), and 72°C (2 min), and then final extension at 72°C for 7 min. The purified and diluted primary PCR product was used as template for nested PCR with one nested gene specific reverse primer (R2) and adaptor walker primer (Table 2). The gene specific primers (R1 and R2) were designed on the basis of chromoplast-specific *lycopene β cyclase *(*CYC-B*) cDNA sequence [GenBank: AF254793] [12]. The secondary PCR product was gel purified, cloned in pDrive vector (QIAGEN) and sequenced.

**Table 2:** Primers used in this study (Incorporated restriction site sequences are underlined)

Primer	Sequence (5'-3')
Walker1	CTA ATA CGA CTC ACT ATA GGG NNN NA TGC
Walker2	CTA ATA CGA CTC ACT ATA GGG NNN NT AGC
Walker3	CTA ATA CGA CTC ACT ATA GGG NNN NG ATC
Walker4	CTA ATA CGA CTC ACT ATA GGG NNN NC TAG
Adaptor walker	CTA ATA CGA CTC ACT ATA GGG
R1	GAT AAT GAT CAC GTC GAA TTG AG
R2	CTC TGG CTT TGA TGT GGGT GCT
D0-F	CCC AAGCTT GAC TTC ACC CTT CTT TCT TGT C
D1-F	CCC AAGCTT GAT CTA CTG GAA ATT GAC CAA C
D2-F	CCC AAGCTT TCA CTA TGT TTG TTA GTG CTT G
D3-F	CCC AAGCTT GAA CAA TTT TCC TCA AAG TTG TAG
D-R	CG GGATCC AGA AAA TGT AAA GAT TGA TAA TGG
CaMV35S-F	GAA TGC TAA CCC ACA GAT GGTTAG
*GUS*-R	GTT CAA CGC TGA CAT CAC CAT TG
*CYC-B*-F	GGG TAA TGA GCC ATA TTT AAG GG
*CYC-B*-R	TAG GAT CCA GAT CAA AGA AAG CG
*EFlα*-F	TGG TCATTG GTC ATG TTG A
*EFlα*-R	GCA GAT CAT TTG CTT GAC ACCAAG

### Analysis of the *ShCYC-B* Promoter Sequences

The 5' flanking sequence upstream to ATG of the *CYC-B *cDNA was searched for known transcription factor binding sites using the PLACE [31] and PlantCARE [32] databases. Transcription start site was predicted by using Neural Network Promoter Prediction softwares [ [30]; http://www.fruitfly.org/seq_tools/promoter.html].

### *ShCYC-B* promoter deletions and promoter-GUS reporter gene constructs

The 908 bp 5' upstream to ATG of the *CYC-B *was considered as full-length *ShCYC-B *promoter in this study and was designated as D0-908. The 5' deletion fragments of D0-908 namely D1-818, D2-578 and D3-436 (D1, D2, and D3 indicates deletions, the numbers after the hypen indicates number of nucleotides upstream to ATG present in the deletion fragment) were amplified with vent^® ^DNA polymerase (New England Biolabs, Beverly, MA) using forward primers (D0-F, D1-F, D2-F, D3-F) with *Hind *III restriction site and one common reverse primer (D-R) with *Bam *HI site at their 5' end (Table 2). The amplified fragments were digested with *Hind *III and *Bam *HI restriction enzyme and cloned in to pBI121 by replacing *CaMV35S *promoter. The pBI121 binary vector was used as positive control (Fig. 3). The binary vectors pBI121, pD0-908 (full-length promoter construct) and pD1-818, pD2-578, pD3-436 (truncated constructs) were transformed into *Agrobacterium *strain LBA4404 by freeze-thaw method. Plasmid was isolated from transformed *Agrobacterium *cultures and was back-transformed into *E. coli *(DH5α) for further confirmation by PCR and restriction analysis of plasmids. The confirmed *Agrobacterium *harboring pBI121, full-length promoter and its deletion fragments driving *GUS *reporter gene were used for transient and stable (transgenic) expression analyses in tomato plants.

### Transient expression assay

Transient expression analysis of *ShCYC-B *promoter was carried out by fruit agroinjection method as described by Orzaez et al [39]. *Agrobacterium *culture (5 mL) harboring pBI121 (P_*CaMV*35*S*_::*GUS *reporter), pD0-908, pD1-818, pD2-578 and pD3-436 were grown overnight from individual colonies at 28°C in YEM medium (pH 6.8) containing kanamycin (50 μg mL^-1^) and rifampicin (25 μg mL^-1^). The culture was then inoculated in to 50 mL induction medium (YEM medium, pH 6.8, supplemented with 20 mM acetosyringone and antibiotics) and grown overnight. Next day, bacterial cells were recovered by centrifugation, resuspended in infiltration medium (10 mM MgCl_2_, 10 mM MES, 200 mM acetosyringone, pH 5.6) and incubated at room temperature with gentle agitation (20 rpm) for 2-3 h. Cultures were agroinjected into green and red fruits of tomato (*S. lycopersicum *var. Pusa Ruby). On third day, non-injected (control) and agroinjected fruits were harvested and transverse sections of the fruit were subjected to GUS histochemical staining as described by Jefferson, [40]. Following the staining, the samples were fixed in 70% (v/v) ethanol.

### Genetic transformation of tomato

Transformation of tomato (*S. lycopersicum *cv. Pusa Ruby) was performed following the method of Cortina and Culianez-Macia [41]. Cotyledons were cut in to 2-3 pieces and pre-cultured for 2 days. The pre-cultured pieces were inoculated with transformed *Agrobacterium *for 15 min and co-cultivated for 2 days. The shoots were regenerated under kanamycin selection (50 μg mL^-1^). The regenerated plants were hardened and then transferred to glass house of National Phytotron Facility, IARI, New Delhi. The presence of the *CYC-B *promoter::*GUS *transgene in the transgenic plants was confirmed by PCR using promoter specific forward (D0-F, D1-F, D2-F, D3-F) and *GUS*-specific reverse primers (Table 2) with genomic DNA extracted from young leaves of T_0 _plants as templates. Fruits from transgenic plants of each construct were examined for *GUS *expression by histochemical analysis. The T_1 _seeds from independent events of each construct were germinated on MS medium supplemented with kanamycin (75 μg mL^-1^) and further screened by PCR analysis using promoter and *GUS*-specific primers as described for T_0 _plants. At least 2-3 independent transgenic events from each construct were examined for stable integration of transgene and its copy number by Southern hybridization (Additional file 1, Figure S1). Single copy transgenic plants were carried further for GUS histochemical assay, quantitative MUG assay and Northern hybridization.

### GUS assay

The histochemical assay and fluorometric assay for *GUS *reporter gene expression were done as described by Jefferson [40] with some modifications. For histochemical GUS analysis, roots, leaves, flower and fruits at different stages of ripening *viz*. early green, mature green, breaker, orange and red ripe, were collected from wild type (*S. lycopersicum *cv. Pusa Ruby) and transgenic plants harboring pBI121*(P*_*CaMV*35*S*_*::GUS *reporter), pD0-908 (full-length *CYC-B::GUS *promoter), pD1-818, pD2-578 and pD3-436 (truncated *CYC-B *promoter::*GUS *constructs) transgenes. The flowers and leaves were immersed as whole, while fruits were cut in transverse sections and incubated in GUS staining solution (0.5 mM X-gluc, 0.1 M NaHPO_4 _pH 8.0, 0.5 mM K_3_Fe(CN)_6_, 0.5 mM K_4_Fe(CN)_6_, 0.01 M EDTA pH8.0, 20% methanol and 0.1% Triton X-100) and incubated overnight at 37°C. After staining, sections were rinsed in 75% ethanol for 2-3 times, and photographed.

Quantitative GUS activity was determined by measuring production of 4-methylumbelliferone (4-MU). For this, samples from 2-3 lines of single copy transgenic lines (two events per construct) were pooled, and used for quantitative MUG assay. Plant tissues were homogenized in 0.4 mL protein extraction buffer (0.1 M NaPO_4_, pH 8.0, 0.1% SDS, 10 mM EDTA,10 mM β-ME, 0.1% Triton X-100) followed by centrifugation at 13,000×*g *4°C for 15 min. The supernatant was used for protein quantification and fluorometric assay. Fifty microliters of supernatant was transferred in to a microcentrifuge tube containing 500 μL GUS reaction buffer (protein extraction buffer containing 10 mM MUG) pre-warmed to 37°C and mixed. One hundred microliters of this mixture was immediately transferred into a 900 μL GUS stop buffer (0.2 M Na_2_CO_3_) to serve as a control. The sample reaction mixture was incubated at 37°C. One hundred microliters of aliquots were removed at 15, 30, 60 and 120 min intervals and mixed with 900 μL GUS stop buffer. GUS activity was measured in fluorometer (VersaFluor, Bio-Rad, Hercules, CA) with excitation at 365 nm and emission at 455 nm. The amount of 4-MU was determined from a standard curve. Protein concentrations of the samples were determined using Bradford reagent (Bio-Rad, Hercules, CA) and BSA as a standard. GUS activity was expressed as pmol 4-MU mg protein^-1 ^min^-1^. Data are presented as the mean (± standard error) of GUS activity from three independent determinations.

### RT-PCR and Northern-blot analyses

Total RNA was isolated from the leaves, flower and fruits at different stages of development from wild type cv. Pusa Ruby (*S. lycopersicum*) and *S. habrochaites *genotype EC520061 using RNeasy^® ^plant mini kit (QIAGEN). Wild type fruits were sampled at green, breaker, orange and red ripe stage. As genotype EC520061 is green fruited, progress of ripening was broadly defined on the basis of seed color and development. The seed color changed from green to light yellow to dark brown therefore stages were defined as green seeded fruit as immature, light yellow seeded as mature green, dark yellow seeded equivalent to breaker, brownish seeded as equivalent to orange and dark brown seeded as ripe stage. For RT-PCR experiments, first-strand cDNA was synthesized from 250 ng of the DNase-treated RNA in a final volume of 20 μL using anchored oligo (dT) of 18-mers according to the manufacturer's instructions (Sensiscript^® ^RT kit, QIAGEN). One microlitre of first-strand cDNA was used as template in PCR for analysis using *CYC-B *gene specific primers. *Elongation factor 1α *(*EF1α*) gene primers (Table 2) were used as internal control in 50 μL reaction volume. The conditions of the PCR were: 94°C for 4 min; 30 cycles of 94°C for 50 s, 59°C for 50 s, 72°C for 50 s; and final extension at 72°C for 5 min. The PCR products were separated on 1.4% agarose gel. To eliminate the possibility of PCR amplification from genomic DNA contamination in RNA samples, a control RT-PCR reaction was carried out without adding reverse transcriptase.

For Northern hybridization, samples from single copy transgenic events were used for total RNA isolation. Wherever required two independent transgenic events carrying single copy of deletion constructs were used for further confirmation of results. About 20-30 μg of total RNA was fractionated on 1.2% (w/v) formaldehyde agarose gel electrophoresis transferred onto Hybond N^+^-Nylon membranes (GE Healthcare) by capillary transfer. After transfer, RNA was fixed by UV-cross linking and hybridized with [α-^32^P] dCTP-labeled *GUS *cDNA fragment corresponding to the entire *GUS *open reading frame. Blots were rehybridized using a [α-^32^P] dCTP-labeled *EFlα *probe as a control for RNA loading.

## Abbreviations

*LYC-B*: Chloroplast-specific *lycopene β-cyclase*; *CYC-B*: Chromoplast-specific *lycopene β-cyclase; *SNP: Single Nucleotide Polymorphism; CAPS: Cleaved Amplified Polymorphic Sequence; MUG: 4-methylumbelliferone glucuronide; GUS: β-glucuronidase; TSS: Transcription Start Site

## Authors' contributions

MD: performed all the experiments. VC assisted with DNA sequence analysis and histochemical GUS analysis. KCB together with MD and VC designed the experiments and wrote the manuscript. All authors read and approved the manuscript.

## Supplementary Material

Additional file 1 **Fig. S1. Representative blots for Southern analysis of T_1 _transgenic plants harboring *CYC-B *full-length promoter and its deletion fragments**. *GUS *coding sequence excised from pBI121 was used as probe; +ve, *GUS *cDNA; WT, wild-type; D0-2-1, D0-4-1, and D0-4-3, T_1 _transgenic lines of full-length promoter; D1-2-1, D1-4-1, D1-4-2 and D1-5-2, T_1 _transgenic plants of D1-818; D2-3-1, D2-3-3, D2-7-1, D2-7-2, D2-8-1 and D2-8-2, T_1 _transgenic plants of D2-578; D3-1-3, D3-2-2, and D3-3-1, T_1 _transgenic plants of D3-436. Click here for file
