# Supplementary figures and images for: Isolation and functional characterization of Lycopene β-cyclase (CYC-B) promoter from Solanum habrochaites
Source: BMC Plant Biol. 2010 Apr 9;10:61. doi: 10.1186/1471-2229-10-61 (PMC2923535; doi:10.1186/1471-2229-10-61)

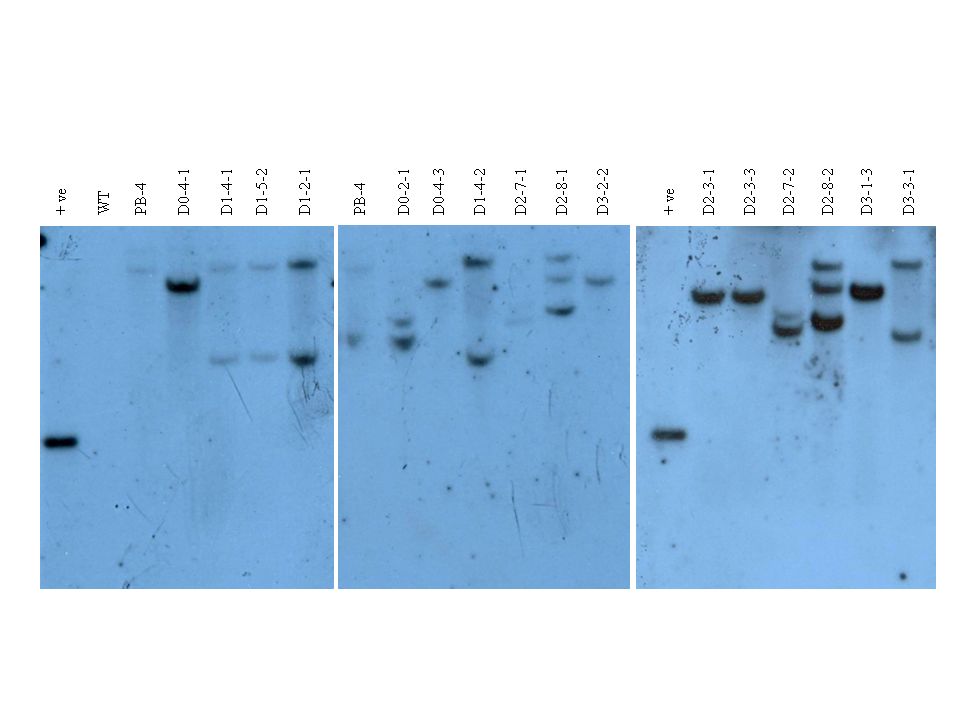

Supplement: Additional file 1 — Fig. S1. Representative blots for Southern analysis of T1 transgenic plants harboring CYC-B full-length promoter and its deletion fragments. GUS coding sequence excised from pBI121 was used as probe; +ve, GUS cDNA; WT, wild-type; D0-2-1, D0-4-1, and D0-4-3, T1 transgenic lines of full-length promoter; D1-2-1, D1-4-1, D1-4-2 and D1-5-2, T1 transgenic plants of D1-818; D2-3-1, D2-3-3, D2-7-1, D2-7-2, D2-8-1 and D2-8-2, T1 transgenic plants of D2-578; D3-1-3, D3-2-2, and D3-3-1, T1 transgenic plants of D3-436. [file 1471-2229-10-61-S1.JPEG]
